# *In Vitro* and *In Vivo* Infectious Potential of *Coxiella burnetii*: A Study on Belgian Livestock Isolates

**DOI:** 10.1371/journal.pone.0067622

**Published:** 2013-06-28

**Authors:** Marcella Mori, Samira Boarbi, Patrick Michel, Raïssa Bakinahe, Katleen Rits, Pierre Wattiau, David Fretin

**Affiliations:** 1 Bacterial Zoonoses of Livestock, Operational Directorate Bacterial Diseases, Veterinary and Agrochemical Research Centre, VAR-CODA-CERVA, Brussels, Belgium; 2 Food borne and Highly Pathogenic Zoonoses and Antibiotic Resistance, Operational Directorate Bacterial Diseases, Veterinary and Agrochemical Research Centre, VAR-CODA-CERVA, Brussels, Belgium; Texas A&M Health Science Center, United States of America

## Abstract

Q-fever is a zoonosis caused by the gram-negative obligate intracellular pathogen *Coxiella burnetii*. Since its discovery, and particularly following the recent outbreaks in the Netherlands, *C. burnetii* appeared as a clear public health concern. In the present study, the infectious potential displayed by goat and cattle isolates of *C. burnetii* was compared to a reference strain (Nine Mile) using both *in vitro* (human HeLa and bovine macrophage cells) and *in vivo* (BALB/c mice) models. The isolates had distant genomic profiles with one - the goat isolate - being identical to the predominant strain circulating in the Netherlands during the 2007–2010 outbreaks. Infective doses were established with ethidium monoazide-PCR for the first time here applied to *C. burnetii*. This method allowed for the preparation of reproducible and characterized inocula thanks to its capacity to discriminate between live and dead cells. Globally, the proliferative capacity of the Nine Mile strain in cell lines and mice was higher compared to the newly isolated field strains. *In vitro*, the bovine *C. burnetii* isolate multiplied faster in a bovine macrophage cell line, an observation tentatively explained by the preferential specificity of this strain for allogeneic host cells. In the BALB/c mouse model, however, the goat and bovine isolates multiplied at about the same rate indicating no peculiar hypervirulent behavior in this animal model.

## Introduction

Q-fever is a zoonosis caused by *Coxiella burnetii*, an intracellular gram-negative bacterium. Q-fever manifests in humans as acute or chronic illness. Acute Q-fever ranges from an asymptomatic state to an abrupt, flu-like illness that can be accompanied with high fever, general malaise, pneumonia, myalgia and hepatitis. Chronic Q-fever occurs in individuals in approximately 1–5% of cases, and typically manifests as endocarditis [1], [2]. Humans contract the disease mainly by inhalation of airborne particles contaminated with *C. burnetii* (feces, milk or birth products -placenta and amniotic fluid-) of infected animals [3], [4]. During abortion, up to 10^9^ *C. burnetii* cells per gram of placenta can be excreted [5]. Considering the infective dose of this bacterium has been reported to be close to one [6], these products are obviously hazardous to humans.

Attention to Q-fever as a potential public health threat increased following the recent outbreaks in the Netherlands. On this occasion, the number of cases notified by the Dutch authorities rose to more than 4,000 between 2007 and 2010, representing a quite unique phenomenon [7]. The outbreak was retrospectively associated with an increase of dairy goat farm density in the affected area. However, because association with farming or other occupational activities could not be documented for all human Q fever cases reported during the outbreaks [8], intrinsic factors (*e.g.* the presence of unusual virulence factors) associated with the circulating isolates have been questioned [7], [9], [10], [11], [12], [13], [14]. Scientific evidence however is missing to clarify this point. Extensive genotypic characterization of human and animal *C. burnetii* isolates demonstrated the polyclonal nature of the Dutch outbreaks [9], [15], [16], [17], [18]. Genetic diversity was limited though, and all isolates could be rooted to one founder genotype named CbNL01 [17] belonging to group G in 6-locus Multiple Loci Variable Number of Tandem Repeats Analysis (MLVA) typing [9], type-1 in single nucleotide polymorphism (SNP) analysis [15] and MST33 in multispacer sequence typing (MST) [18]. In Belgium, investigations were subsequently conducted in order to assess whether this particular strain was disseminating from the neighboring country, the Netherlands, and to evaluate its potential threat for the human population and the agricultural sector.

*C. burnetii* is a small pleomorphic γ-proteobacterium with an intracellular life style. Following internalization by a host cell, *C. burnetii* replicates in a large acidophilic parasitophorous vacuole [19]. Upon changes in the surrounding conditions, the organism undergoes peculiar developmental cycles resulting in a differentiated stage called the large cell variant, which starts replicating exponentially. The replicating phase is followed by a maturation step that leads to a stable *C. burnetii* form, the small cell variant that is somewhat similar to an endospore in its ability to resist harsh environmental conditions [19].

The infectious potential of *C. burnetii* is also associated with the structure of its lipopolysaccharide (LPS), which consists in either the virulent phase I (LPS I) or the avirulent phase II (LPS II) [20]. Bacteria in phase I are characterized by a full-length LPS and cause Q-fever in humans or a similar disease in experimental animal models. Upon serial passages in cell cultures or repeated injections in embryonated eggs, virulent *C. burnetii* undergo a shift to the phase II form characterized by a non-reversible shortening of the LPS molecule [21]. This transition proceeds through gradual reduction of the polysaccharidic part of the LPS, which in turn is accompanied by large genomic deletions [20]. Phase II bacteria are avirulent and do not replicate in immuno-competent hosts.

Because of its intracellular life-cycle, considerable constraints are associated with the laboratory handling of *C. burnetii* and to the establishment of accurately documented *in vitro* and *in vivo* infection protocols. The infectious potential of *C. burnetii* has been investigated with cell lines [22], [23] and animal models, such as guinea pigs [24], [25], [26], [27] and mice [28]. While guinea pigs seem particularly susceptible to *C. burnetii* infection in aerosol models, the use of mice provides researchers with the benefits of the numerous genetic and biological resources engineered for this model animal to study candidate virulence factors expressed by a given pathogen or to assess the efficacy of candidate vaccines. In many studies, however, repeatability and strain to strain reproducibility are often questionable due to the methodology used to characterize the bacterial inocula. Often the methodology fails in taking into consideration the proportion of live/dead cells (*e.g.* quantitative PCR-qPCR-based methods) or lacks sensitivity (*e.g.* microscopic counting of infection foci in cell monolayers).

In this study, we applied *in vitro* and *in vivo* infection models to compare the proliferative capacity of the reference Nine Mile strain in phase I (NMI) with that exhibited by two *C. burnetii* field strains isolated from infected Belgian animals. The latter consisted in one strain isolated from a cow and another one isolated from a goat imported from the Netherlands and displaying a genotypic profile identical to the main *C. burnetii* strain circulating during the 2007–2010 Dutch outbreaks. We used ethidium monoazide (EMA)-PCR to determine the exact amount of living organisms in the inocula used to infect cultured cell lines or BALB/c mice. The monitored parameters were the proliferative capacity measured by real-time PCR quantification of the number of *C. burnetii* genome-equivalents over time, the organs’ weight and the serological response.

## Materials and Methods

### Cell Lines and Culture Media

Human carcinoma HeLa cells were cultivated in Dulbecco modified Eagle medium (DMEM) supplemented with 10% foetal calf serum (FCS) and 0.1 mM of nonessential amino acids solution (all from Life Technologies, Carlsbad, CA). SV40-transformed bovine macrophage cells (SV40 macrophages) were maintained in RPMI1640 medium supplemented with 10% FCS and 2 mM L-glutamine (Life Technologies).

### Animals

Specific-pathogen-free (SPF) 6-week old female OF1 and BALB/c mice were purchased from Charles River experimental animal facility (Charles Rivers, Wilmington, MA). Mice were housed in SPF isolated cages (Tecniplast, Buguggiate, Italy) at the L3 animal facility in CODA-CERVA under controlled conditions. Experiments conducted in this study were all approved by the Animal Ethical Committee of CODA-CERVA (Project Approval No. RF 10/6228) and performed in L3 containment.

### Isolation of *C. burnetii* Strains from Infected Animal Samples

*C. burnetii* strains from animals suspected of infection by the Q fever agent were isolated by a two-step protocol including: (i) an *in vivo* isolation procedure in OF1 mouse strain (Charles Rivers), in which 200 µl of homogenized abortion material or bulk milk were injected intraperitoneally (i.p.) in 7 week-old female mice at day 1 (first challenge) and day 21 (second challenge). Spleens were harvested at day 32 and analyzed for the presence of *C. burnetii* by real-time qPCR as previously described [29]. Only those samples displaying a Ct value <25 were kept for processing in the second step, (ii) which consisted in *C. burnetii* amplification in seven-day old embryonated chicken eggs obtained by injection in the yolk sac of 100 µl of a 1∶10 dilution of the spleen homogenate in phosphate buffered saline (PBS) from the previous step. Egg embryos dying before day 5 were discarded. Depending on egg lifespan and not later than one week after injection, yolk sacs were collected, washed twice in physiological water (Biorad, La Jolla, CA) and homogenated mechanically using the ULTRA-TURRAX Tube Drive system (IKA, Staufen, Germany). DNA was extracted from 200 µl of this homogenate and 1/50^th^ of the DNA preparation was analyzed for the presence of *C. burnetii* by real-time qPCR [29]. Only those samples displaying a Ct value <20 were used to infect cell lines and BALB/c mice.

### *C. burnetii* Genotyping on Clinical Samples and on Amplified Cultures

DNA was extracted from 200 µl of *C. burnetii*-infected embryonated egg homogenates or directly from suspect animal samples with the Qiagen DNA mini kit (Qiagen, Hilden, Germany), according to the manufacturer’s instructions. MLVA was performed on the isolated DNA for 10 markers (MS03, MS21, MS22, MS30, MS36, MS27, MS28, MS31, MS24, and MS34) [9], [30], [31]. Amplified fragments were analyzed on a CEQ 8000 Genetic Analysis System (Beckman Coulter, Indianapolis, IN). Single-nucleotide genotyping was performed exactly as described [15].

### Ethidium Monoazide (EMA)-PCR and Real Time qPCR

Yolk sacs from *C. burnetii*-infected embryonated egg were homogenized in PBS buffer before analysis by EMA-PCR. Working dilutions were those described in the results section. EMA (Geniul, Spain) was added to a final concentration of 100 µM. Samples were then incubated for 30 minutes at 4°C in the dark and vortexed regularly. Subsequently, they were exposed to visible light for 30 minutes in an appropriate instrument (PhAST blue system, Geniul). DNA was isolated from 175 µl of EMA-treated samples with MagMax beads (Life Technologies) as described by the manufacturer. One tenth of the recovered DNA sample was used in qPCR analysis according to a real-time PCR procedure targeting *com1* and published by Kersh *et al.* [32]. For quantitative measurements (qPCR), a calibration curve was built up by determining the threshold cycle (Ct) for each serial dilution of a reference sample of our own consisting of a highly concentrated *C. burnetii* suspension. When dilutions were plotted on a graph in logarithmic scale as a function of Ct values, a calibration curve was obtained. This curve was transformed into an absolute quantification curve by adjusting the curve parameters with the Ct determined on an external reference (LSI) containing precisely 40 genome equivalents per µl.

### *In vitro* Infection Protocol

Cells were suspended in infection medium (DMEM for HeLa cells or RPMI 1640 for SV40 macrophages, both supplemented with 5% FCS) and infected separately with ca. 10^6^ ‘living’ *C. burnetii* organisms by adding adjusted dilutions of the embryonated egg homogenates (Multiplicity of Infection = 100). After 24 hours incubation (37°C, 5% CO_2_), infected cells were washed three times in 200 µl culture medium. After the third washing, 200 µl fresh medium supplemented with 50 µg/ml Gentamicin (Life Technologies) was added to the wells. Cells were harvested from four individual wells for each time point by adding distilled water (100 µl/well), transferred into 1.5-ml screw capped tubes and heated for 30 min at 82°C. The number of *C. burnetii* genome equivalents was established with the real-time PCR targeting *com1*. Uninfected control cells were kept spatially close to infected cells and were always found negative in qPCR, ruling out possible cross-contamination. Growth kinetics of *C. burnetii* were established on 1 × 10^4^ HeLa cells or SV40 macrophages cultivated in 96-well tissue culture plates.

### *In vivo* Infection Protocol

Six-week old female BALB/c mice (Charles River) were injected i.p. with 10^4^ *C. burnetii* cells (200 µl) categorized as ‘living’ by EMA-PCR conducted on the same day. Dilutions of *C. burnetii* from stock suspensions were obtained in physiological water (Biorad). Control mice were injected with physiological water only. A group of five mice was sacrificed at each time point for each strain. Serum, spleen and lungs were collected for further analysis (Fig.S1). Overall, 20 mice were enrolled for each strain study.

### ELISA Quantification of mice anti-*C. burnetii* IgM and IgG

Mice IgM and IgG antibody levels directed against *C. burnetii* were quantified following adaptation of a commercially available ELISA kit (Ruminant Q Fever – Serum/Milk ELISA kit, LSI, Lisieux, France). Analyses were conducted according to the manufacturer’s instructions with the following modifications. One hundred µl of diluted mouse serum were deposited on plates supplied with the kit, coated with *C. burnetii* whole-cell antigens, and incubated for 1 hour at 37°C. Optimal mice serum dilution and anti-mouse conjugate dilution were determined following two-way titrations conducted on both negative samples originating from uninfected animals and positive (*C. burnetii* infected) samples (Fig. S2). Maximal absorbance within the linear range occurred when mouse serum was diluted 1∶20 and all subsequent analyses were performed using serum samples diluted 1∶20. For mouse IgM quantification, peroxidase-conjugated goat polyclonal anti-mouse IgM (AbD Serotec, Oxford, UK) was found to best perform at 1∶500 dilution. For mouse IgG quantification, the protein-G based kit’s conjugate was used at 1∶100 dilution. Optical densities were read out at 490 nm on a microplate reader (Perkin Elmer Wallac Victor, Santa Clara, MA). The robustness of this mouse-adapted commercial ELISA was thoroughly validated through repeated measurements taken in independent assays.

### Bioinformatics and Statistical Analyses

Genetic relatedness of the MLVA profiles was analyzed by Minimum spanning tree using the Bionumerics software package (Applied Maths, Belgium). GraphPad Prism version 6 (GraphPad Software, San Diego, CA) was used to perform two-way analysis of variance test (ANOVA) with post-test in order to validate strain to strain and time-point variations determined with a significance level set to p<0.05.

## Results

### Isolation and Genotypic Characterization of Two *C. burnetii* Field Strains

**Figure 1:**
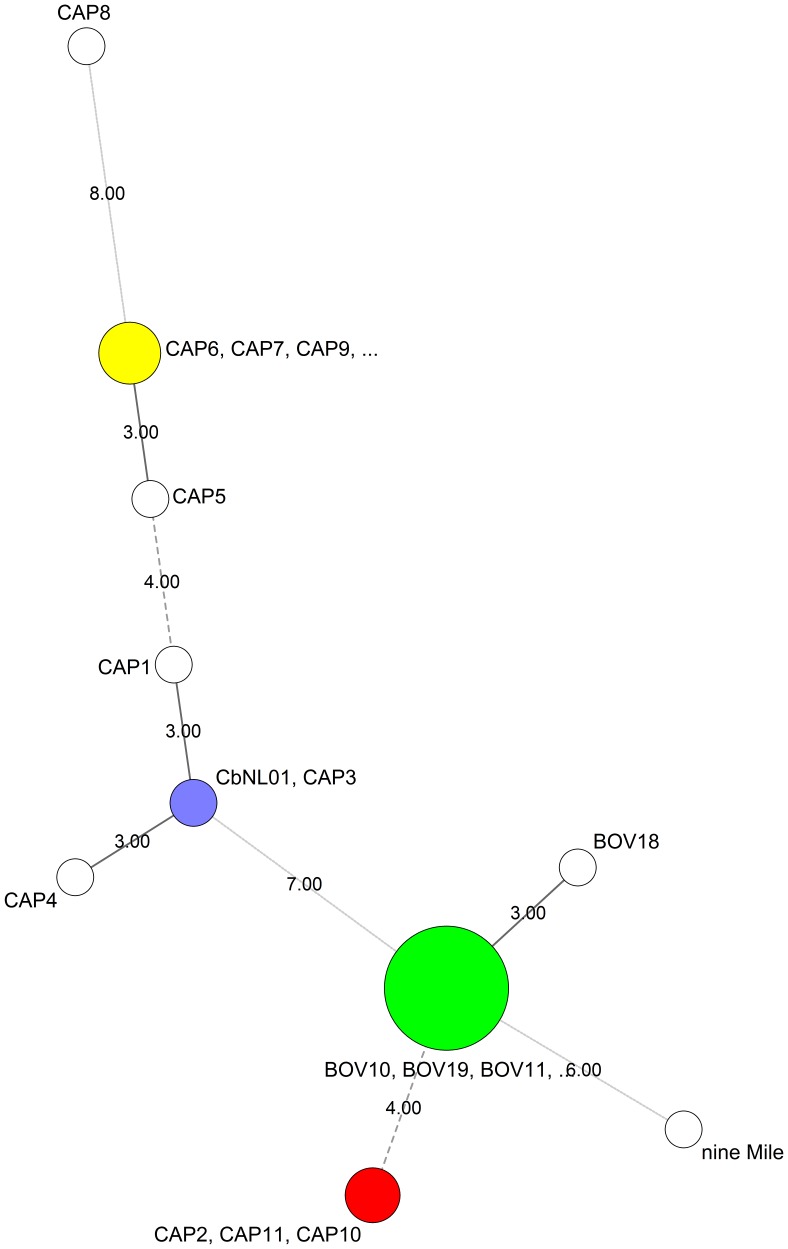
Minimum spanning tree analysis of MLVA profiles from Belgian livestock clinical samples positive for *C. burnetii*. 10-locus MLVA profiles were determined for 11 goat and 20 bovine positive samples collected between 2009 and 2011. Clustering by minimum spanning tree was performed with Bionumerics and included the Nine Mile reference strain and CbNL01 (MLVA profiles derived from publicly available data). Circles outline the genetic profiles of strains found in positive clinical samples. Numbers on the connecting lines refer to the number of markers differing between samples. The size of the circles is proportional to the number of strains bearing the same genetic profile. Most bovine strains (19/20) are closely related. One goat sample (CAP3) was infected with a strain displaying a genetic profile identical to that of CbNL01.

Following large screening programs involving goat and dairy cattle in Belgian farms in 2009–2011, a number of field samples suspected of *C. burnetii* infection were collected. A total of 11 goat and 20 bovine farms were found positive and therefore analyzed. These samples contained sufficient bacterial loads for partial genetic analysis by 10-locus MLVA. Minimum spanning tree analysis of the genetic profiles obtained for the bovine samples (Fig. 1) showed consistent genetic homogeneity, with only one marker varying among those successfully amplified (average of 9.4/10±0.7). In contrast, *C. burnetii* strains from goat samples were of greater variability. Genetic profiles associated with *C. burnetii* infecting goats were not found in cattle, and vice-versa, providing indications for a discrete, possibly species-specific lineage within cattle herds. Among goat samples, we found one (named CAP3 in Fig. 1) displaying a genetic profile identical to the main strain of the Dutch 2007–2010 outbreaks (named CbNL01). This sample was collected in a farm close to the Netherlands’ border in goats imported from this country (as attested by the sanitary label found on the animal ear). From the 31 positive samples, two (CAP3 and BOV1) were kept for further bacterial amplification and characterization (Fig. 1). Isolation was first obtained in OF1 mice as described in Materials and Methods. The complete genotype of these strains was obtained by both MLVA and a single nucleotide polymorphism typing scheme [9], [30], [31] and were compared to that of the reference strain Nine Mile phase I (NMI). Results confirmed the genetic diversity observed on raw clinical samples and the high level of identity between the CAP3 goat sample and CbNL01 (Table 1). These strains, from here on named CbBEC1 (the goat strain) and CbBEB1 (the bovine strain), were studied for their infectious potential using *in vitro* cell cultures and a mouse infection model.

**Table 1:** List of *C. burnetii* isolates used in this study.

Sample name	Name of the strain	Country of origin	Source	SNP genotype	MLVA marker and number of repeats									
					03	21	22	28	24	30	31	34	27	36
	CbNL01	The Netherlands	Goat, humans	1	7	6	6	3	11	5	3	7	3	13
CAP3	CbBEC1	Belgium	Goat	1	7	6	6	3	11	5	3	7	3	13*
BOV1	CbBEB1	Belgium	Cattle	2	6	6	6	6	14	6	3	9	2	4
	NMI	U.S.	Tick	3	7	6	6	6	27	6	5	5	4	4

country of origin, source of isolation, SNP and MLVA genotypes. The goat-derived strain (CbBEC1) has been isolated from an animal imported from the Netherlands. This strain has the same genetic profile as the CbNL01, the *C. burnetii* founder strain of the 2007–2010 Dutch outbreak episodes. SNP and MLVA profile of CbNL01 was derived from publicly available data.

* approximated value.

### Enumeration of Viable *C. burnetii* Cells by EMA-PCR

**Figure 2:**
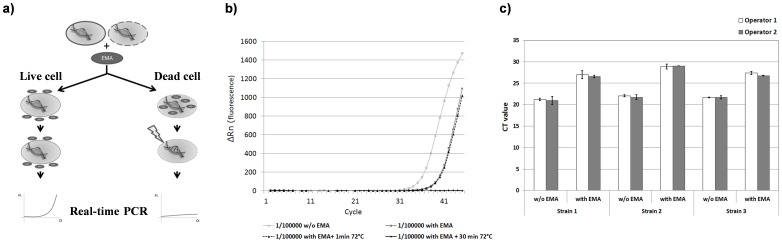
Use of EMA-PCR for enumerating living *C. burnetii* cells. Ethidium monoazide (EMA)-PCR was used to count live *C. burnetii* cells. A) Schematic representation of the EMA-PCR principle. EMA is unable to diffuse through intact bacterial cells with a non-damaged cell wall. Therefore, subsequent detection of those cells by qPCR is not hindered (left side). Following diffusion inside permeable cells, EMA creates covalent cross-links in the DNA molecule upon exposure to a blue-light source. DNA originating from those cells will not be amplified by qPCR (right side). B) EMA-PCR on dead *C. burnetii* bacterial cells. *C. burnetii* bacterial cells killed by heating at various time points and treated with EMA stain failed to yield positive PCR reactions. C) Repeatability and reproducibility of EMA-PCR measurements. Two operators using the same protocol and the same three stock-aliquots of *C. burnetii* determined the bacterial load in independent measurements. To assess intra-assays repeatability, five measurements were collected for each time point.

Because *C. burnetti* is an obligate intracellular pathogen, inocula can only be characterized by indirect enumeration methods. We therefore applied ethidium monoazide (EMA) qPCR to define the ratio of live/dead *C. burnetii* in the inocula used for *in vitro* and *in vivo* infections. The principle of the test is briefly outlined in Fig2a. Calibration curves were established on serial dilutions of a *C. burnetii* DNA stock solution by amplifying the single-copy gene *com1* (see Materials & Methods). To assess the potential of EMA-PCR in discriminating live and dead cells, we inactivated *C. burnetii*-containing samples diluted 1∶10^5^ (corresponding roughly to 10^6^ cells/ml) by heating at 72°C for 30 minutes. After this treatment, PCR amplification of *C. burnetii* DNA was undetectable within 45 PCR cycles, demonstrating template DNA inactivation by EMA and confirming the capacity of the technique to differentiate dead from living cells (Fig. 2b). Reproducibility and repeatability of the technique were evaluated by testing each of three different strains five times by two different operators (Fig. 2c). Overall, results demonstrated the robustness of the EMA-PCR approach for characterizing *C. burnetii* inocula both quantitatively and qualitatively. To ensure that repeated freeze/thaw cycles did not affect the viability of *C. burnetii* stock samples, EMA-PCR was conducted on each inoculum prior to infection on the day of the experiment.

### Infectious Potential of *C. burnetii* Strains in Cell Line Models

**Figure 3:**
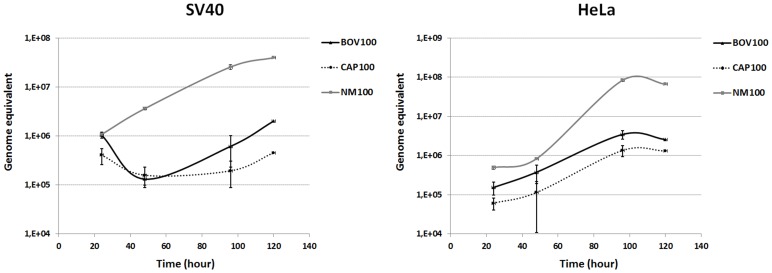
Growth curves of NMI, CbBEB1 and CbBEC1 in HeLa and SV40 cells. Cells were infected at 100 M.O.I. for 24 hours. Infected cells were then washed and grown in gentamicin-containing medium for 96 additional hours. At fixed time-points, deionized water was added and cells lysed by a 30-min incubation at 82°C. *C. burnetii* loads were quantified by qPCR on 1∶15 dilutions. Each point indicates the average of four independent cultures and its corresponding standard deviation.

To characterize the proliferative capacity of the two Belgian field strains (CbBEC1-goat and CbBEB1-bovine) compared to the NMI reference, *C. burnetii* growth curves were established on SV40-transformed bovine macrophages and on human HeLa cells. CbBEC1 and CbBEB1 cultures passed 1 time on mice and 2 times on embryonated eggs, were incubated for 24 h with the above cultured cells at 100 M.O.I. Bacterial loads (genomic equivalent) were then quantified by qPCR at several time points post-infection (p.i.). Results are shown in figure 3 for four independent cultures. Overall, NMI showed the fastest growth rate characterized by a 37-fold increase (p<0.0001) in cell counts at 120 h p.i. when infecting SV40-transformed bovine macrophages. The growth rate of NMI in HeLa cells was even faster with a 170-fold increase (p<0.0001) in cell counts at 120 h p.i. (Fig. 3). Field strains CbBEC1 and CbBEB1 displayed equal proliferative capacity in HeLa cells, characterized by a 22-fold increase in cell counts at 96 h p.i. In bovine macrophages, however, CbBEB1 (Fig. 3 right panel) replicated 5 times faster than CbBEC1 (15-fold increase in cell counts at 120 h p.i. for CbBEB1, 3-fold increase for CbBEC1, p>0.005). These results are indicative of a preferential replication of the bovine *C. burnetii* isolate in allogenic (macrophage) cell lines.

### Infectious Potential of Three *C. burnetii* Strains in BALB/c Mice

**Figure 4:**
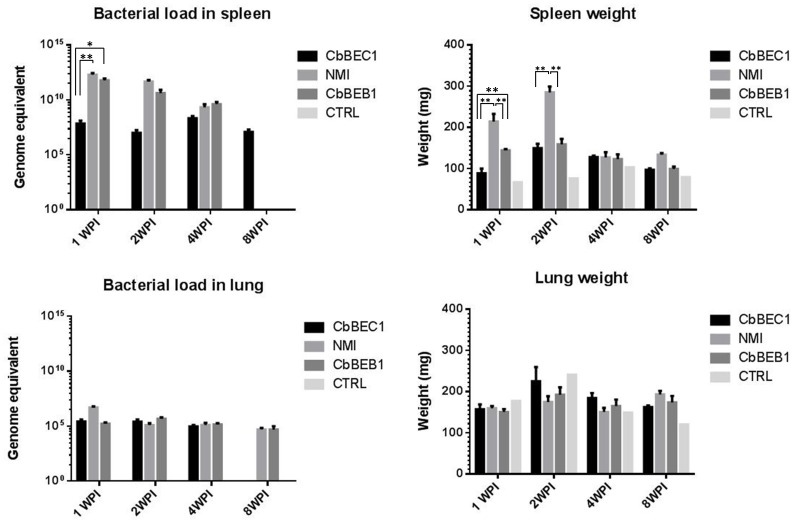
*C.burnetti* colonization of spleen and lungs as evaluated by qPCR quantification and organ weight. Graphs show *C. burnetii* loads in spleen and lungs (left panels) or variations in spleen and lungs weight (right panels). Results are given for uninfected mice and mice infected with NMI, CbBEB1 and CbBEC1 at 1, 2, 4 and 8 weeks post infection. Mean values +/− Standard Error (SE) for 5 mice are shown. * indicates p-values smaller than 0.05 ** indicates p-values smaller than 0.01.

**Figure 5:**
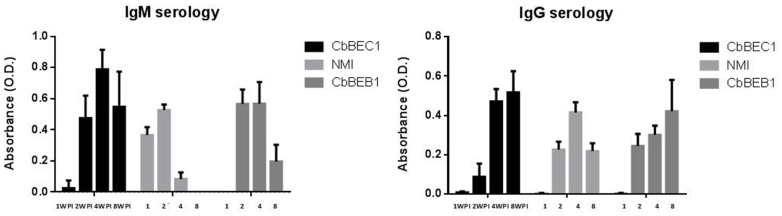
Mice anti-*C. burnetii* IgM and IgG assayed by ELISA at various time-points. IgM (left panel) and IgG (right panel) average levels of anti-*C. burnetii* antibodies as measured by ELISA in NMI, CbBEB1 and CbBEC1 infected mice at 1, 2, 4 and 8 weeks post-infection. Optical density values (O.D.) were normalized with values from uninfected control mice. Each value shown is the mean value determined on 5 mice +/− SE.

Because *in vitro* infection models unlikely account for the complexity of the mechanisms associated with *C. burnetii* pathogenesis, we also analyzed the infectious potential of NMI, CbBEC1 and CbBEB1 *in vivo* in a mouse model. Groups of five female BALB/c mice were injected i.p. with 10^4^ *C. burnetii* cells. Spleen, lung and serum were collected 1, 2, 4 and 8 weeks p.i. and bacterial loads, organ weights and antibody responses were analyzed. Bacterial colonization of the spleen was transiently higher for the NMI and the CbBEB1 strains as compared to CbBEC1. This difference was mostly significant 1 week p.i. and characterized by a 3×10^4^ fold and a 8×10^3^ fold increase in *C. burnetii* counts for NMI and CbBEB1, respectively (Fig. 4 upper-left panel). However, bacterial loads in the spleen decreased with time and both NMI and CbBEB1 were completely cleared from the spleen 8 weeks p.i. (Fig. 4 upper-left panel). An increase in spleen weight was noticed in mice 1 week p.i. with NMI and CbBEB1. Spleens from animals infected with the latter two strains were significantly bigger than those from uninfected (control) or CbBEC1-infected mice at both 1 and 2 weeks p.i. (NMI) or at 1 week p.i. only (CbBEB1) (Fig. 4 upper-right panel). No significant correlation between bacterial load and spleen weight could be observed at any time in individual mice (see supplemental Table S1). Colonization of the spleen by the CbBEC1 strain was lower but constant in time. The latter strain was not completely cleared from the spleen 8 weeks p.i. (Fig. 4 upper-left panel). Colonization of the lung was minimal for the three strains, and no significant difference was observed in terms of bacterial load or organs weight (Fig. 4 lower panels). At the serological level, IgG and IgM titers were similar for all three strains (Fig. 5). Differences were observed in the associated kinetics, though. IgM titers peaked at 2 weeks p.i. for NMI, 2–4 weeks p.i. for CbBEB1, 4 weeks p.i. for CbBEC1and decreased afterwards (Fig. 5 left panel). IgG responses were maximal at 4 weeks p.i for NMI and 8 weeks p.i. for CbBEB1 and CbBEC1 (Fig. 5 right panel). Altogether, NMI was thus the most actively replicating strain, followed by CbBEB1 and CbBEC1. Serologically, the three strains elicited similar antibody responses with different kinetics. Globally, the field-derived strains showed similar infective behavior in BALB/c mice.

## Discussion

The Dutch outbreaks that took place between 2007 and 2010 were unprecedented in the history of Q fever in three aspects. First, these outbreaks were the largest ever recorded with more than 4,000 human cases [33]. Second, related to that, international scientific awareness (and funding) has been brought back to this neglected zoonosis. Third, unprecedented amounts of prospective data were generated at human and veterinary levels to shed light on the etiology, pathology and biology of *C. burnetii* [33]. Several genotypes of *C. burnetii* could be identified during the Dutch outbreaks, but genetic diversity was limited and could be rooted back to a major clone termed CbNL01. Circulation of highly infectious *C. burnetii* strains has been suggested but not demonstrated so far [17].

In this work, we studied the infectious potential of two Belgian field isolates by using *in vitro* and *in vivo* models of infection and comparing it to that of the NMI reference strain. One strain included in our study displayed a genetic profile undistinguishable from that of the Dutch CbNL01 strain (as defined by MLVA and SNP typing). Our study also reports the use of EMA-PCR applied for the first time to characterize the live/dead ratios of *C. burnetii* cells in the inocula used for experimental infection models.

In the absence of an experimental procedure adequately modeling a human Q fever infection in the laboratory [27], cell culture infection and mouse infection by the intra-peritoneal route are provisional options offering plasticity and flexibility to study several aspects of *C. burnetii* pathogenic potential. Before starting the work with infection models, we questioned current methodologies aimed at determining infective *C. burnetii* doses. We first looked for a protocol able to discriminate between live and dead bacteria. Quantitation of total *C. burnetii* loads in infectious inocula can be obtained by quantitative PCR through real-time amplification of the single-copy gene *com1* [32], [34]. This measurement is appropriate when one single strain is used in the study [35]. However, this method does not provide any information regarding the viability of the detected bacteria, a critical parameter that is traditionally measured through cell culture infections of serially diluted samples [28]. The latter method is tedious and lacks sensitivity. Combining qPCR and EMA dye staining, such as reported here, turns out to be an effective means to achieve viability characterization. EMA selectively penetrates damaged bacterial cells and, upon photolysis, inhibits the PCR amplification of genomic DNA from dead cells, proving direct measurement of intact (living) cells. EMA-PCR is widely used as viability test for various pathogens [36], [37], [38], [39] and it was here successfully applied to *C. burnetii*. In our hands, EMA-PCR was found robust and reproducible when performed within well-defined dilution limits (from 1∶100 in our setting). Non-specific chicken DNA originating from embryonated eggs competes with bacterial DNA in binding EMA dye and is likely to explain the failure of the method to discriminate between live and dead *C. burnetii* cells at higher sample concentration. The developed procedure specifically applies to the embryonated egg matrix, and needs to be adapted for use on other matrices such as animal tissues or biological fluids.

The NMI reference strain replicated more efficiently than the field strains in both our *in vitro* and *in vivo* infection models, an observation that can be explained either by a higher infectious potential of NMI *per se* [6], [41], or a higher adaptation of this strain to *in vitro* cultures as compared to field strains. In order to exclude differences associated with alterations induced by *in vitro* culturing, the field strains were processed the same way (one passage on mice and twice on embryonated eggs). Because *in vitro* adaptation in non immuno-competent hosts might lead to irreversible changes in the LPS structure shifting from the virulent phase I to the avirulent phase II [20], the number of culture passages in embryonated eggs was kept to a minimum.

The Belgian bovine isolate replicated faster than the goat isolate in SV40-transformed bovine macrophages, although both grew slower that the NMI reference in this cell line. The difference was more significantly marked at later time points, ca. 5 days post infection as the probable consequence of the quite long doubling time of fresh *C. burnetii* field strains in cell cultures [40]. This observation, together with the apparent genetic homogeneity observed for bovine *C. burnetii* isolates, suggests that host-specificity might exist within *Coxiella* strains, as it does for other intracellular pathogens [42]. It has been shown recently that bovine milk products derived from various European countries contained *C. burnetii* DNA displaying highly similar genetic profiles as assessed by 6-locus MLVA [43]. This homogeneity in profiles also resembled that observed in the bovine isolates reported in the present work. Another study, using whole-genome microarrays, compared the genome content of 52 *C. burnetii* isolates retrieved from hard ticks, mammals and humans, demonstrated the existence of a single conserved genomotype for hard tick isolates. [44].

The *in vivo* behavior of *C. burnetii* has been studied in a variety of animal models. We chose to use BALB/c mice because of the intermediate level of sensitivity of this mouse strain to the Q fever agent and its successful use by several research groups in the past [45]. In our intra-peritoneal infection model, colonization of BALB/c mice by *C. burnetii* was observed in all tested organs, albeit at low level in lungs. Colonization of the spleen was a good indicator of infectivity and persistence over time, allowing individual *C. burnetii* strains to be compared to the NMI reference. In contrast with previous works [34], [45], correlation between splenic bacterial loads and spleen weight could not be observed by us at individual mouse level. NMI proliferated faster than the two field strains. Clearance from the spleen was observed for NMI and CbBEB1 but not for CbBEC1, which persisted 8 weeks post infection. The latter strain also elicited delayed IgM and IgG responses compared to the two other strains. Our *in vitro* experiments together with the BALB/c infection model showed that CbBEB1 and CbBEC1 proliferative capacity in the considered hosts are similar. In spite of the fact that one of the used field strain was highly similar - if not identical - to the Dutch outbreak strain CbNL01, no hypervirulent behavior could be demonstrated in mice.

## Supporting Information

Figure S1 **Schematic representation of the protocol used for** ***in vivo*** **infection in BALB/c mice.** 6- week old female BALB/c mice were injected i.p. with 10^4^ *C. burnetii* living bacterial cells as defined by EMA-PCR. Mice were housed in independent SPF cages for the uninfected control and the infected animals. Groups of five mice were sacrificed 1, 2, 4 and 8 weeks post infection for each strain and for uninfected controls. Spleen, lungs and blood were collected for further analyses.
(TIFF)

Figure S2 **Adaptation of a commercially available ELISA kit for mouse anti-** ***C. burnetii*** **IgM and IgG quantification.** Optimal serum dilution and secondary antibody complex/conjugate concentrations was investigated by two-way titration. Titrations were conducted on negative samples (NC-dashed lines) derived from uninfected animals or from positive samples (PC-full lines) originating from *C. burnetii* infected mice. The applied conditions were as follows: serum dilution 1∶20, anti-mouse IgM conjugate 1∶500, kit’s anti-IgG conjugate 1∶1000.
(TIFF)

Table S1 **Data on spleen weight (mg) and associated bacterial load (genome equivalent) obtained from individual mice for each of** ***C. burnetii*** **isolates used in this study.**
(XLSX)
