# Supplementary figures and images for: In Vitro and In Vivo Infectious Potential of Coxiella burnetii: A Study on Belgian Livestock Isolates
Source: PLoS One. 2013 Jun 28;8(6):e67622. doi: 10.1371/journal.pone.0067622 (PMC3695903; doi:10.1371/journal.pone.0067622)

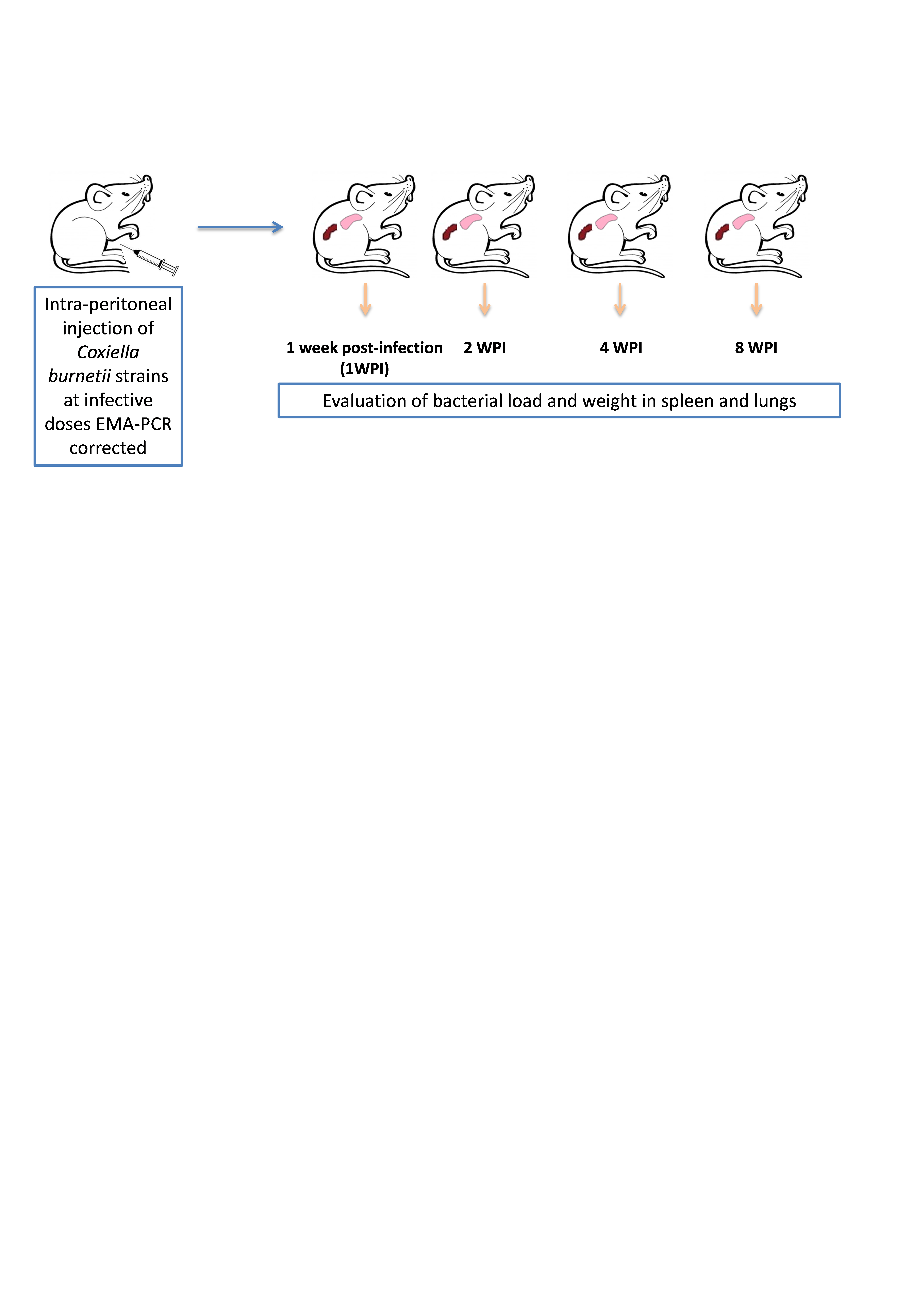

Supplement: Figure S1 — Schematic representation of the protocol used for in vivo infection in BALB/c mice. 6- week old female BALB/c mice were injected i.p. with 104 C. burnetii living bacterial cells as defined by EMA-PCR. Mice were housed in independent SPF cages for the uninfected control and the infected animals. Groups of five mice were sacrificed 1, 2, 4 and 8 weeks post infection for each strain and for uninfected controls. Spleen, lungs and blood were collected for further analyses. (TIFF) [file pone.0067622.s001.tiff]

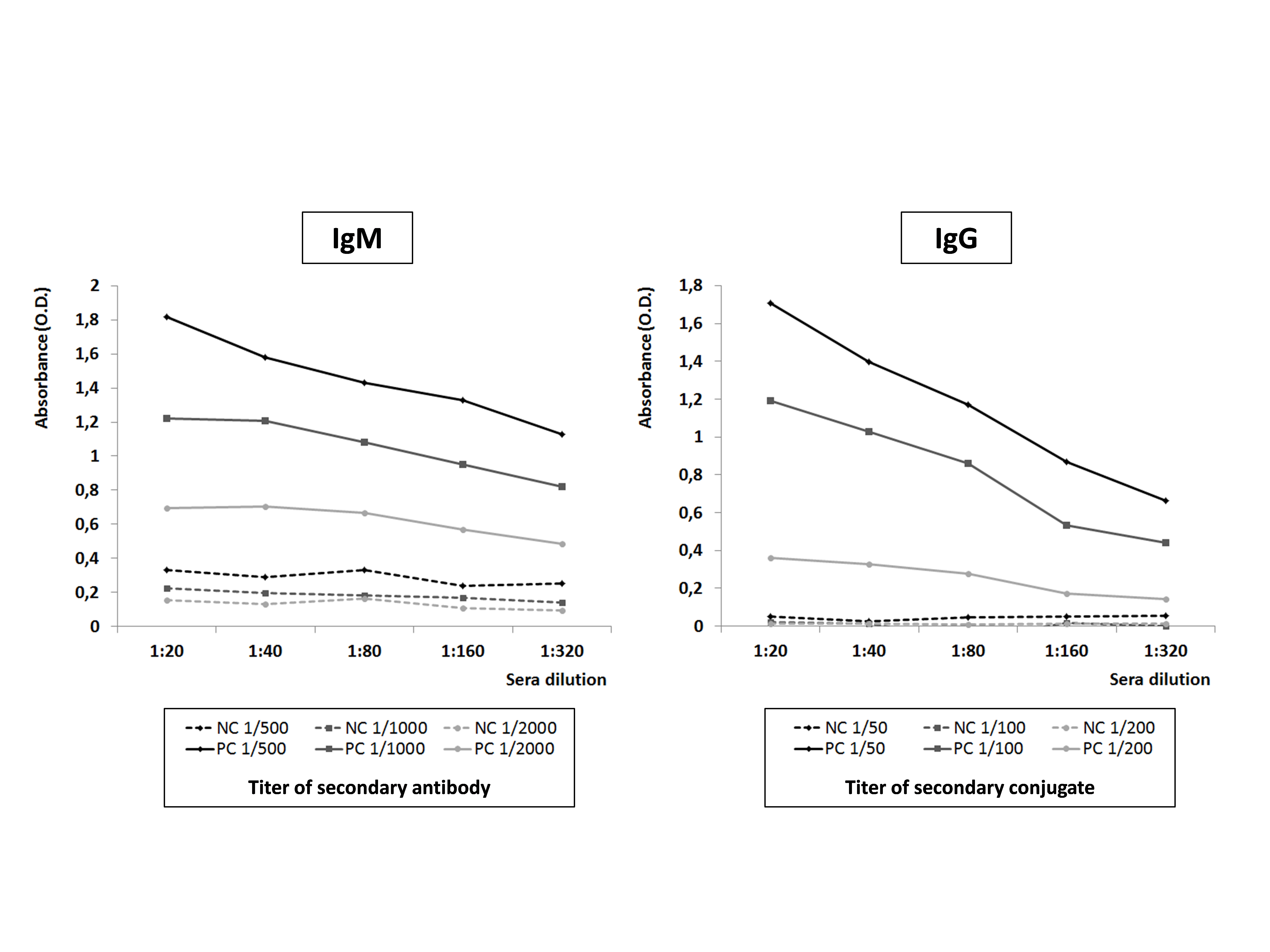

Supplement: Figure S2 — Adaptation of a commercially available ELISA kit for mouse anti- C. burnetii IgM and IgG quantification. Optimal serum dilution and secondary antibody complex/conjugate concentrations was investigated by two-way titration. Titrations were conducted on negative samples (NC-dashed lines) derived from uninfected animals or from positive samples (PC-full lines) originating from C. burnetii infected mice. The applied conditions were as follows: serum dilution 1∶20, anti-mouse IgM conjugate 1∶500, kit’s anti-IgG conjugate 1∶1000. (TIFF) [file pone.0067622.s002.tiff]
